# Supplementary material for: Respiratory virus behavior: Results of laboratory-based epidemiological surveillance
Source: PLoS One. 2024 Oct 3;19(10):e0307322. doi: 10.1371/journal.pone.0307322 (PMC11449343; doi:10.1371/journal.pone.0307322)
Supplement: S1 File — (DOCX) [file pone.0307322.s001.docx]

**Supporting Information.**

**S1. Sequence and working concentration of primers and probes.**

The primers and probes used for the afore mentioned diagnoses.

| **Respiratory virus** | **Primer/probe** | **Primer/probe sequence** |
| --- | --- | --- |
| Adenovirus | ADV-F | GCCCCAGTGGTCTTACATGCACATC |
|  | ADV-R | GCCACGGTGGGGTTTCTAAACTT |
|  | ADV-P | [FAM]TGCACCAGACCCGGGCTCAGGTACTCCGA[BHQ-1] |
| Human Bocavirus | BCV-F | TGC AGA CAA CGC YTA GTT GTT T |
|  | BCV-R | CTG TCC CGC CCA AGA TAC A |
|  | BCV-P | CCA GGA TTG GGT GGA ACC TGC AAA |
| Coronavirus HKU1 | CRV HKU1-F | CCTTGCGAATGAATGTGCT |
|  | CRV HKU1-R | TTGCATCACCACTGCTAGTACCAC |
|  | CRV HKU1-P | [FAM]TGTGTGGCGGTTGCTATTATGTTAAGCCTG[BHQ-1] |
| Coronavirus OC43 | CRV OC43-F | CGATGAGGCTATTCCGACTAGGT |
|  | CRV OC43-R | CCTTCCTGAGCCTTCAATATAGTAACC |
|  | CRV OC43-P | [FAM]TCCGCCTGGCACGGTACTCCCT[BHQ-1] |
| Coronavirus NL63 | CRV NL63-F | GACCAAAGCACTGAATAACATTTTCC |
|  | CRV NL63-R | ACCTAATAAGCCTCTTTCTCAACCC |
|  | CRV NL63-P | [FAM]AACACGCTT[BHQ-1]CCAACGAGGTTTCTTCAACTGAG |
| Coronavirus 229E | CRV 229E-F | CAGTCAAATGGGCTGATGCA |
|  | CRV 229E-R | AAAGGGCTATAAAGAGAATAAGGTATTCT |
|  | CRV 229E-P | [FAM]CCCTGACGACCACGTTGTGGTTCA[BHQ-1] |
| Human Metapneumovirus | MNV-F | CAA GTG TGA CAT TGC TGA YCT RAA |
|  | MNV-R | ACT GCC GCA CAA CAT TTA GRA A |
|  | MNV-P | TGG CYG TYA GCT TCA GTC AAT TCA ACA GA |
| Enterovirus | ETV-F | GGTGGCTGCGTTGGC |
|  | ETV-R | GAAACACGGACACCCAAAGTA |
|  | ETV-P | [FAM]TCCTCCGGCCCCTGAATGYGGC[BHQ-1] |
| Rhinovirus | RNV-F1 | C(P)[A]GCC[T]GCGTGGC |
|  | RNV-F2 | C(P)[A]GCC[T]GCGTGGT |
|  | RNV-R | GAAACACGGACACCCAAAGTA |
|  | RNV-P | [FAM]TCCTCCGGCCCCTGAATGYGGC[BHQ-1] |
| Sincicial Respiratorio | VSR-F | GGCAAATATGGAAACATACGTGAA |
|  | VSR-R | TCTTTTTCTAGGACATTGTAYTGAACAG |
|  | VSR-P | [FAM]CTGTGTATGTGGAGCCTTCGTGAAGCT[BHQ-1] |
| Parainfluenza 1 | PIV 1-F | ACAAGTTGTCAAYGTCTTAATTCRTAT |
|  | PIV 1-R | TCGGCACCTAAGTARTTYTGAGTT |
|  | PIV 1-P | [FAM]ATAGGCCAAAGAT[BHQ-1]TGTTGTCGAGACTATTCCAA |
| Parainfluenza 2 | PIV 2-F | GCATTTCCAATCTACAGGACTATGA |
|  | PIV 2-R | ACCTCCTGGTATAGCAGTGACTGAAC |
|  | PIV 2-P | [FAM]CCATTTACCT[BHQ-1]AAGTGATGGAATCAATCGCAAA |
| Parainfluenza 3 | PIV 3-F | TGGYTCAATCTCAACAACAAGATTTAAG |
|  | PIV 3-R | TACCCGAGAAATATTATTTTGCC |
|  | PIV 3-P | [FAM]CCCRTCTGT[BHQ-1]TGGACCAGGGATATACTACAAA |
| Parainfluenza 4 | PIV 4-F | CTGCCAAATCGGCAATTAAAC |
|  | PIV 4-R | CTGGACGCAATCATAAGRTGATTC |
|  | PIV 4-P | [FAM]CATTATTATCTCTGCT[BHQ-1]TTCCTTACAGGCCACATCA |

The primers were rehydrated at 10 micromolar while the probes were rehydrated at 5 micromolar, preparing a reaction mixture according to the following:

| **Reactive** | **Volume in microliters** |
| --- | --- |
| Molecular biology grade water | 5.0 |
| Primer-F | 0.5 |
| Primer-R | 0.5 |
| Probe | 0.5 |
| Agpath-ID Enzyme, One-step RT-PCR | 1.0 |
| Buffer 2X | 12.5 |
| Final volume per sample | 20.0 |

The amplification program was as follows:

| **Retro transcription** | **DNA polymerase activation** | **Denaturation** | **Hybridization, polymerization, Fluorescence emission** |
| --- | --- | --- | --- |
| 1 cicle  45C/10:00 min | 1 cicle  95C/10:00 min | 45 cicles  95C/00:10 segundos | 45 cicles  55C/01:00 min |
