# Supplementary material for: Respiratory virus behavior: Results of laboratory-based epidemiological surveillance
Source: PLoS One. 2024 Oct 3;19(10):e0307322. doi: 10.1371/journal.pone.0307322 (PMC11449343; doi:10.1371/journal.pone.0307322)
Supplement: Attachment — Submitted filename: Answers.docx [file pone.0307322.s002.docx]

**Journal requirements:**

When submitting your revision, we need you to address these additional requirements.

**1. Please ensure that your manuscript meets PLOS ONE's style requirements, including those for file naming. The PLOS ONE style templates can be found at**

<https://journals.plos.org/plosone/s/file?id=wjVg/PLOSOne_formatting_sample_main_body.pdf>

and

<https://journals.plos.org/plosone/s/file?id=ba62/PLOSOne_formatting_sample_title_authors_affiliations.pdf>

A = The changes were made.

**2. We suggest you thoroughly copyedit your manuscript for language usage, spelling, and grammar. If you do not know anyone who can help you do this, you may wish to consider employing a professional scientific editing service.**

A = The changes were made. We send it to American Journal Experts (Letter from AJE is included as an "Other" file).

Whilst you may use any professional scientific editing service of your choice, PLOS has partnered with both American Journal Experts (AJE) and Editage to provide discounted services to PLOS authors. Both organizations have experience helping authors meet PLOS guidelines and can provide language editing, translation, manuscript formatting, and figure formatting to ensure your manuscript meets our submission guidelines. To take advantage of our partnership with AJE, visit the AJE website (<http://learn.aje.com/plos/>) for a 15% discount off AJE services. To take advantage of our partnership with Editage, visit the Editage website ([www.editage.com](http://www.editage.com/)) and enter referral code PLOSEDIT for a 15% discount off Editage services.  If the PLOS editorial team finds any language issues in text that either AJE or Editage has edited, the service provider will re-edit the text for free.

Upon resubmission, please provide the following:

The name of the colleague or the details of the professional service that edited your manuscript

A copy of your manuscript showing your changes by either highlighting them or using track changes (uploaded as a *supporting information* file)

A clean copy of the edited manuscript (uploaded as the new *manuscript* file)

**3. When completing the data availability statement of the submission form, you indicated that you will make your data available on acceptance. We strongly recommend all authors decide on a data sharing plan before acceptance, as the process can be lengthy and hold up publication timelines. Please note that, though access restrictions are acceptable now, your entire data will need to be made freely accessible if your manuscript is accepted for publication. This policy applies to all data except where public deposition would breach compliance with the protocol approved by your research ethics board. If you are unable to adhere to our open data policy, please kindly revise your statement to explain your reasoning and we will seek the editor's input on an exemption. Please be assured that, once you have provided your new statement, the assessment of your exemption will not hold up the peer review process.**

A = Repository: 10.6084/m9.figshare.24895014

**4. Please review your reference list to ensure that it is complete and correct. If you have cited papers that have been retracted, please include the rationale for doing so in the manuscript text, or remove these references and replace them with relevant current references. Any changes to the reference list should be mentioned in the rebuttal letter that accompanies your revised manuscript. If you need to cite a retracted article, indicate the article’s retracted status in the References list and also include a citation and full reference for the retraction notice.**

A = Citations recommended by the reviewers were included and adhered to the journal's guidelines.

**Reviewers' comments:**

Reviewer's Responses to Questions

**Comments to the Author**

1. Is the manuscript technically sound, and do the data support the conclusions?

The manuscript must describe a technically sound piece of scientific research with data that supports the conclusions. Experiments must have been conducted rigorously, with appropriate controls, replication, and sample sizes. The conclusions must be drawn appropriately based on the data presented.

Reviewer #1: Yes

Reviewer #2: Yes

2. Has the statistical analysis been performed appropriately and rigorously?

Reviewer #1: Yes

Reviewer #2: I Don't Know

3. Have the authors made all data underlying the findings in their manuscript fully available?

The [PLOS Data policy](http://www.plosone.org/static/policies.action#sharing) requires authors to make all data underlying the findings described in their manuscript fully available without restriction, with rare exception (please refer to the Data Availability Statement in the manuscript PDF file). The data should be provided as part of the manuscript or its supporting information, or deposited to a public repository. For example, in addition to summary statistics, the data points behind means, medians and variance measures should be available. If there are restrictions on publicly sharing data—e.g. participant privacy or use of data from a third party—those must be specified.

Reviewer #1: Yes

Reviewer #2: Yes

4. Is the manuscript presented in an intelligible fashion and written in standard English?

PLOS ONE does not copyedit accepted manuscripts, so the language in submitted articles must be clear, correct, and unambiguous. Any typographical or grammatical errors should be corrected at revision, so please note any specific errors here.

Reviewer #1: Yes

Reviewer #2: Yes

5. Review Comments to the Author

Please use the space provided to explain your answers to the questions above. You may also include additional comments for the author, including concerns about dual publication, research ethics, or publication ethics. (Please upload your review as an attachment if it exceeds 20,000 characters)

**Reviewer #1**.

In general, the authors described the epidemiological behavior of several common respiratory viruses other than influenza virus and SARS-CoV-2 in Mexico, according to multiplex PCR laboratory-based epidemiological surveillance.

These are my comments:

From the editorial point of view, the article flow and path was reasonable, however, I encountered several typo mistakes and grammar and writing issues throughout the text. Please double-check the whole text for revision.

- The title reflects the manuscript content.

- Abstract is adequate, describing the message of the experiments.

- Background: is adequately described.
**The sentence: “Likewise, the adenovirus regains importance due to the mass vaccination with the biologicals that have been administered in Mexico to combat COVID-19, some of which contain adenovirus as a vector”. The authors mean that COVID-19 adenovirus vector-included vaccines caused to bring up this disease? Is it an exact issue or assumption? Please explain.**

A = This is an assumption: “Likewise, the adenovirus regains importance due to the mass vaccination with the biologicals that have been administered in Mexico to combat COVID-19, some of which contain adenovirus as a vector; ***that would probably confer protection for the adenovirus***”. It was decided to delete the paragraph to avoid confusion.

- M&M: is reproducible.
**Authors have not mentioned about the RNA extraction kit they used. If it covers all kind of RNA and DNA viruses? Also, any cDNA synthesis was performed before multiplex PCR?**
A = This section was added.

**Laboratory analysis of samples.**

The SuperScript™ III Platinum® One-Step RT‒qPCR System Kit (Invitrogen, Carlsbad, California, USA. Catalog: 12574035) was used with a 7500 Fast Real-Time PCR System (Applied Biosystems®, Foster City, California, USA) to amplify viral genetic material. Primers and probes were used for each of the following viruses: metapneumovirus, respiratory syncytial virus, human parainfluenza viruses 1–4, betacoronavirus 1, human coronavirus (OC43, HKU1, 229E, NL63), rhinovirus, enterovirus, adenovirus and bocavirus. The human RNAse P (RP) gene was used as an internal control. The samples were evaluated in uniplex reactions with the following reaction mixture: 12.5 μL of 2x reaction mix, 0.5 μL of each primer and probe, 1 μL of enzyme, 5.5 μL of RNase-free water, and 5 μL of total nucleic acid. The following thermocycling conditions were used: one cycle of 45°C for 10 min and 95°C for 10 min and 45 cycles of 95°C for 15 sec and 55°C for 1 min. DNA or RNA lyophilizates (AmpliRun® Vircell; Granada–Spain) were used as the positive controls for all evaluated viruses. Samples that presented amplification for any of the viral markers plus the internal control were considered positive. Samples without amplification of the viral markers but with amplification of the internal control were considered negative.

- Results are described in proper way.
**As there are age groups of infants and youngsters it is better to mention male and female instead of man/men and woman/women.**

A = The changes were made.

Figure 1 is too blurred and the range of colors is too close to distinct. Please change the color range.
A = The changes were made.

Discussion: It is nicely organized and presented on the data justified, compared with other similar literatures.
**In the part “The frequency by type of respiratory virus, other than influenza or SARS-CoV-2, was similar to the one presented in other parts of the world.[12,13,14,15,16]” …There are also some similar studies conducted in the middle east (Iran) that may add value to the context of the manuscript (PMID: 38093303, PMID: 36482963).**
A = The changes were made. Suggested reference added.

The frequency by type of respiratory virus, other than influenza or SARS-CoV-2, was similar to the one presented in other parts of the world.[12-16] Yet, it contrasts with a study carried out in China during the COVID-19 pandemic, since between 2020 and 2021, the results for adenovirus decreased considerably, [17] also in Iran adenovirus had the lowest prevalence (0.1%) whereas in our 2020 study compared to the previous year an increase was witnessed, which was stressed in 2022.

**As I mentioned in introduction, I was concerned regarding adenovirus prevalence information. Authors should make a strong justification to relate the adenovirus prevalence to the COVID-19 adenovirus vector-included vaccines.**
A = It was decided to delete the paragraph to avoid confusion.

**- References are up to date, just double-check for the consistency.**
A = The changes were made.

Overall comments:
The scientific content of the manuscript is really interesting and worth reporting for the health care system comparing the prevalence of several respiratory viruses all over the world after double-checking the whole text for the writing errors and applying corrections with critical consideration.
It has merit for publication after minor modification.

**Reviewer #2.**

Summary of the manuscript:
This observational study aimed to characterize the epidemiological behavior of respiratory viruses from 2017 to 2023, with a focus on samples beyond SARS-CoV-2 due to increased interest during the COVID-19 pandemic. Using laboratory-based epidemiological surveillance, the study analyzed 4,632 samples through multiplex PCR. The most prevalent viruses were respiratory syncytial virus (26.3%) and rhinovirus (24.8%), primarily affecting children under 5 years. Parainfluenza virus 4 showed an age-adjusted odds ratio of 4.1 for death. In conclusion, respiratory syncytial virus and rhinovirus were common, while parainfluenza virus 4 was associated with fatalities.

Comments
**1. There are already several reports as provided below to inform that such kind of work is already reported and that reduces the novelty of the current submission.
Reporting after Covid-19
Pedroza-Uribe IM, Vega Magaña N, Muñoz-Valle JF, Peña-Rodriguez M, Carranza-Aranda AS, Sánchez-Sánchez R, Venancio-Landeros AA, García-González OP, Zavala-Mejía JJ, Ramos-Solano M, Viera-Segura O and García-Chagollán M (2024) Beyond SARS-CoV-2: epidemiological surveillance of respiratory viruses in Jalisco, Mexico. Front. Public Health. 11:1292614. doi: 10.3389/fpubh.2023.1292614
Reporting before Covid-19
Fernandes-Matano L, Monroy-Muñoz IE, Angeles-Martínez J, Sarquiz-Martinez B, Palomec-Nava ID, Pardavé-Alejandre HD, Santos Coy-Arechavaleta A, Santacruz-Tinoco CE, González-Ibarra J, González-Bonilla CR, Muñoz-Medina JE. Prevalence of non-influenza respiratory viruses in acute respiratory infection cases in Mexico. PLoS One. 2017 May 3;12(5):e0176298. doi: 10.1371/journal.pone.0176298. PMID: 28467515; PMCID: PMC5415110.**
A = The changes were made. Suggested reference added.

**2. Samples collected are not classified in terms of area and timelines. Demographic distribution of the samples used in the report may help further understanding.**

A = Data is added in the study to complement this aspect. We added Figure 2.

**3. As per the reported timelines of the survey, “Surveillance of respiratory viruses in Mexico has been carried out through the 2009-2020 Influenza Epidemiological Surveillance System and the Viral Respiratory Disease Epidemiological Surveillance System (ERV according to its initials in Spanish) (from 2020 to date) [10] as a non-probabilistic sampling of negative results for influenza or SARS-CoV-2.”. The reason for taking samples from 2017-2023 is not specified or justified.**
A = Added text adding explanation.

Surveillance of respiratory viruses in Mexico has been carried out through the 2009-2020 Influenza Epidemiological Surveillance System^[9]^ and the Viral Respiratory Disease Epidemiological Surveillance System (ERV according to its initials in Spanish) (from 2020 to date) ^[10]^ as a non-probabilistic sampling of negative results for influenza or SARS-CoV-2; in both periods with different operational case definitions, but in both in a syndromatic manner to identify mild and severe cases of acute respiratory disease. In this sense, samples were analyzed from years prior to the COVID-19 pandemic, as well as during it.

**4. Most of the samples are from Paediatric cohort and hence there are issues of variable susceptibility. The analysis may also be done in different groups to distinguish among the overall samples. Alternatively, only one cohort, i.e. paediatric may be considered for total duration from 2009 to till date in order to make the work and report a bit unique and a systematic one.**

A = Data is available from 2017. Likewise, the protocol submitted for evaluation by the Committee was approved for the period from 2017 to 2023.

6. PLOS authors have the option to publish the peer review history of their article ([what does this mean?](https://journals.plos.org/plosone/s/editorial-and-peer-review-process#loc-peer-review-history)). If published, this will include your full peer review and any attached files.
If you choose “no”, your identity will remain anonymous but your review may still be made public.
**Do you want your identity to be public for this peer review?** For information about this choice, including consent withdrawal, please see our [Privacy Policy](https://www.plos.org/privacy-policy).

Reviewer #1: No

Reviewer #2: **Yes:** Dr Vijay Nema
