# Supplementary material for: Respiratory virus behavior: Results of laboratory-based epidemiological surveillance
Source: PLoS One. 2024 Oct 3;19(10):e0307322. doi: 10.1371/journal.pone.0307322 (PMC11449343; doi:10.1371/journal.pone.0307322)
Supplement: Attachment — Submitted filename: Answers.docx [file pone.0307322.s003.docx]

**PONE-D-23-43288R1**

**Respiratory virus behavior: results of laboratory-based epidemiological surveillance.**

PLOS ONE

Dear Dr. Cabrera Gaytán,

**Reviewers' comments:**

Reviewer's Responses to Questions

**Comments to the Author**

1. If the authors have adequately addressed your comments raised in a previous round of review and you feel that this manuscript is now acceptable for publication, you may indicate that here to bypass the “Comments to the Author” section, enter your conflict of interest statement in the “Confidential to Editor” section, and submit your "Accept" recommendation.

Reviewer #1: All comments have been addressed

Reviewer #3: (No Response)

2. Is the manuscript technically sound, and do the data support the conclusions?

The manuscript must describe a technically sound piece of scientific research with data that supports the conclusions. Experiments must have been conducted rigorously, with appropriate controls, replication, and sample sizes. The conclusions must be drawn appropriately based on the data presented.

Reviewer #1: Yes

Reviewer #3: Yes

3. Has the statistical analysis been performed appropriately and rigorously?

Reviewer #1: I Don't Know

Reviewer #3: Yes

4. Have the authors made all data underlying the findings in their manuscript fully available?

The [PLOS Data policy](http://www.plosone.org/static/policies.action#sharing) requires authors to make all data underlying the findings described in their manuscript fully available without restriction, with rare exception (please refer to the Data Availability Statement in the manuscript PDF file). The data should be provided as part of the manuscript or its supporting information, or deposited to a public repository. For example, in addition to summary statistics, the data points behind means, medians and variance measures should be available. If there are restrictions on publicly sharing data—e.g. participant privacy or use of data from a third party—those must be specified.

Reviewer #1: No

Reviewer #3: Yes

5. Is the manuscript presented in an intelligible fashion and written in standard English?

PLOS ONE does not copyedit accepted manuscripts, so the language in submitted articles must be clear, correct, and unambiguous. Any typographical or grammatical errors should be corrected at revision, so please note any specific errors here.

Reviewer #1: Yes

Reviewer #3: Yes

6.Review Comments to the Author

Please use the space provided to explain your answers to the questions above. You may also include additional comments for the author, including concerns about dual publication, research ethics, or publication ethics. (Please upload your review as an attachment if it exceeds 20,000 characters).

**Reviewer #1:**

The authors have addressed most of the comments. There are just 3 things to address before acceptance:

**1- Figure 1 is still blurring and I think if the authors cannot correct it, Journal editorial office may help to make it sharp.**

A = The change request was made.

**2- Also I noticed some changes in the height of bars in new Figure 1 compared to old one. If the authors have made any changes in sample number and statistical analysis it is substantial to be mentioned to Editor.**

A = The observation is correct and pertinent. We realized that there was an error when selecting the virus data over time; that is why they were modified. We apologize for this.

**3- In the new paragraph under “Laboratory analysis of samples” title; please add information about the RNA extraction kit used. If it covers all kind of RNA and DNA viruses?**

A = We added lines and “S1. Sequence and working concentration of primers and probes”.

**Reviewer #3**:

This study analyzed laboratory-based respiratory virus surveillance data from 2017 to 2023 in Mexico, before and during the COVID-19 pandemic. Similar studies on respiratory virus infections have been published nationally and regionally.

However, this study has strengths in analyzing laboratory test results from patients of all ages in Mexico, excluding influenza and COVID-19 infections, to analyze the national respiratory virus epidemic pattern over time.

In laboratory-based epidemiological surveillance, authors can discuss hypotheses and inferences about differences in characteristics by age and non-clinical type of respiratory virus, and the possibility of co-infection, or suggest the possibility of follow-up research.

*My opinions are as follows:*

**- Indicate whether surveillance data from IESS (Influenza Epidemiological Surveillance System) and ERV (Respiratory Virus Epidemiology) are methodologically the same, and whether there are differences in period and surveillance system.**

A = Both are the same, in 2020 the name was changed and new COVID-19 variables were included (see line 88-89).

**- The increase in ADV for children under 5 years of age presented in lines 189-190 is not clearly visible due to resolution issues in Figure 1.**

A = The observation is correct; the statement was removed.

**- Since there was no epidemic of pediatric hepatitis caused by ADV, the reason for analyzing ADV and co-infections in Table 3 is unclear.**

A = We eliminated Table 3.

**- The reason for analyzing enterovirus together with respiratory viruses is not specified.**

A = Enterovirus is included in the RT-qPCR (respiratory virus) diagnostic panel (FilmArray™).

**- Explanation of the respiratory disease diagnosis algorithm in the Results section and clarification of the 1.6% of other respiratory viruses mentioned in Table 3 are needed.**

A = An explanation was included in the paragraph. The value provided is not linked to table 3. Also we eliminated that table.

**- In the time series representation in Figure 1, adding years along with the months as separate lines on the x-axis from 2017 to January 2023 improves data interpretation.**

A = The change request was made.

**- Indicating in the table captions the statistical methods used in Tables 6, 7, 8, and 9 will improve interpretation of the data. These are logistic regression analysis, multivariate logistic regression analysis, and multivariate analysis that fit the table.**

A = The request to add the type of analysis was included.

**- Reference 29 is about human coronaviruses, but the comparison with parainfluenza viruses and its relevance to the literature on parainfluenza virus mortality needs to be clarified a little more.**

A = The lines 285-294 were modified and the reference.

*Minor terminology needs correction.*

**- Combine the two periods at the end of line 92 into one.**

A = The change request was made.

**- Line 177: Ensure consistency of decimal places by maintaining one decimal place.**

A = The change request was made.

**- Line 217: In the Results section, it is recommended to use "95%IC" instead of "95%CI" in Table 8 to be consistent with Tables 6, 7, and 9.**

A = The change request was made.

**- Curiosity: I'm curious what the "a" in "1a4year" in all tables represents.**

A = An apology for the carelessness in the translation. The change request was made.

**- Lines 167, 174, 175, 176: For consistency, use commas for numbers over 1000.**

A = The change request was made.

**- Including line 173, it appears that the "O" for the OC43 subtype appears as the number 0 throughout the journal.**

A = The change request was made in the manuscript and tables.

**- Clarification is needed regarding the mention of betacoronavirus 1, which is not the focus of this study, in line 132.**

A = The change request was made.

**- Line 182: Check the number of patients for “86”.**

A = The change request was made.

**- Consider expressing the unit as ‘fatality rate (%)’ in Table 5.**

A = The change request was made. In the same way, it is homologated to a decimal.

**- Check once more the calculated values of 34.5% for the entire sample in line 269 and 18.8-36.2% in line 273.**

A = The lines were modified.

7. PLOS authors have the option to publish the peer review history of their article ([what does this mean?](https://journals.plos.org/plosone/s/editorial-and-peer-review-process#loc-peer-review-history)). If published, this will include your full peer review and any attached files.
If you choose “no”, your identity will remain anonymous but your review may still be made public.
**Do you want your identity to be public for this peer review?** For information about this choice, including consent withdrawal, please see our [Privacy Policy](https://www.plos.org/privacy-policy).

Reviewer #1: No

Reviewer #3: No

While revising your submission, please upload your figure files to the Preflight Analysis and Conversion Engine (PACE) digital diagnostic tool, <https://pacev2.apexcovantage.com/>. PACE helps ensure that figures meet PLOS requirements. To use PACE, you must first register as a user. Registration is free. Then, login and navigate to the UPLOAD tab, where you will find detailed instructions on how to use the tool. If you encounter any issues or have any questions when using PACE, please email PLOS at [figures@plos.org](mailto:figures@plos.org). Please note that Supporting Information files do not need this step.
